# Electron Transport in Naphthalene Diimide Derivatives

**DOI:** 10.3390/ma14144026

**Published:** 2021-07-19

**Authors:** Jaroslaw Jung, Arkadiusz Selerowicz, Paulina Maczugowska, Krzysztof Halagan, Renata Rybakiewicz-Sekita, Malgorzata Zagorska, Anna Stefaniuk-Grams

**Affiliations:** 1 Department of Molecular Physics, Faculty of Chemistry, Lodz University of Technology, Zeromskiego 116, 90-924 Lodz, Poland; arkadiusz.selerowicz@dokt.p.lodz.pl (A.S.); paulina.maczugowska@dokt.p.lodz.pl (P.M.); krzysztof.halagan@p.lodz.pl (K.H.); anna.stefaniuk.grams@gmail.com (A.S.-G.); 2 Faculty of Mathematics and Natural Sciences, School of Sciences, Institute of Chemical Sciences, Cardinal Stefan Wyszynski University in Warsaw, Wóycickiego 1/3, 01-815 Warsaw, Poland; r.rybakiewicz@uksw.edu.pl; 3 Faculty of Chemistry, Warsaw University of Technology, Noakowskiego 3, 00-664 Warsaw, Poland; malgorzata.zagorska@pw.edu.pl

**Keywords:** naphhthalene diimide derivatives, HOMO and LUMO levels, DFT calculations, electron-only devices, electron mobility

## Abstract

Two naphthalene diimides derivatives containing two different (alkyl and alkoxyphenyl) N-substituents were studied, namely, *N*,*N*′-bis(*sec*-butyl)-1,4,5,8-naphthalenetetracarboxylic acid diimide (NDI-s-Bu) and *N*,*N*′-bis(4-n-hexyloxyphenyl)-1,4,5,8-naphthalenetetracarboxylic acid diimide (NDI-4-n-OHePh). These compounds are known to exhibit electron transport due to their electron-deficient character evidenced by high electron affinity (EA) values, determined by electrochemical methods and a low-lying lowest unoccupied molecular orbital (LUMO) level, predicted by density functional theory (DFT) calculations. These parameters make the studied organic semiconductors stable in operating conditions and resistant to electron trapping, facilitating, in this manner, electron transport in thin solid layers. Current–voltage characteristics, obtained for the manufactured electron-only devices operating in the low voltage range, yielded mobilities of 4.3 × 10^−4^ cm^2^V^−1^s^−1^ and 4.6 × 10^−6^ cm^2^V^−1^s^−1^ for (NDI-s-Bu) and (NDI-4-n-OHePh), respectively. Their electron transport characteristics were described using the drift–diffusion model. The studied organic semiconductors can be considered as excellent candidates for the electron transporting layers in organic photovoltaic cells and light-emitting diodes

## 1. Introduction

Arylene diimides, studied over 100 years [1,2], in the past century were predominantly used as dyes and pigments (for example, [3,4,5]). They are characterized by the high diversity of their optical properties since their absorption spectra can be tuned either via increasing their aromatic core size or through functionalization with appropriate substituents. They can also be used as luminophores [6]. Grafting of alkyl substituent to imide nitrogen improves their processability, facilitating their deposition in the form of thin layers via solution processing, including printing techniques [7,8,9].

The development of organic electronics in the past 2 decades renewed the research interest in this family of compounds. It was demonstrated that their aromatic core functionalization with electron-withdrawing groups yielded semiconductors suitable for the fabrication of n-channel field organic effect transistors (OFETs) stable in operating conditions [10,11,12]. Core functionalization with electron-donating groups renders diimides ambipolar, suitable for ambipolar OFETs [13]. Redox and electronic properties of arylene diimides can also be tuned through N-functionalization, albeit to a lesser extent [14,15].

An interesting feature of functionalized arylene diimides is their capability of directional crystallization. For example, application of the zone casting technique in the fabrication of n-channel OFETs rendered devices exhibiting significantly improved electron mobilities as compared to those in which spin-coating or dip-coating were used [16,17] Arylene diimides were found to form monolayers on appropriate substrates which exhibited extended 2D supramolecular order, as evidenced by scanning tunneling microscope investigations [15].

Another interesting application of arylene diimides involved bulk heterojunction-type organic photovoltaic cells where they served as the acceptor phase, replacing fullerene derivatives, typically used for this purpose [18,19,20]. There are also reports where the rylene diimide derivative was used as an electron transport layer in organic light-emitting diodes and photovoltaic cells [21].

Two naphthalene diimide derivatives presented in this research differ in the nature of N-substituents (aliphatic vs. aromatic), and for this reason, they show different ionization potential (IP) and electron affinity (EA) values. Their redox, spectroscopic properties, as well as their crystal structure, were already discussed in our previous papers in view of their application in n-channel OFETs [17,22]. This paper is devoted to the detailed elucidation of the electrical transport mechanisms in thin layers of these compounds, complementing the already published data. A detailed analysis of experimental current–voltage characteristics with the use of drift–diffusion current and space charge limited current models will be presented.

## 2. Materials and Methods

### 2.1. Materials and Fabrication of the Test Devices

**Figure 1:**
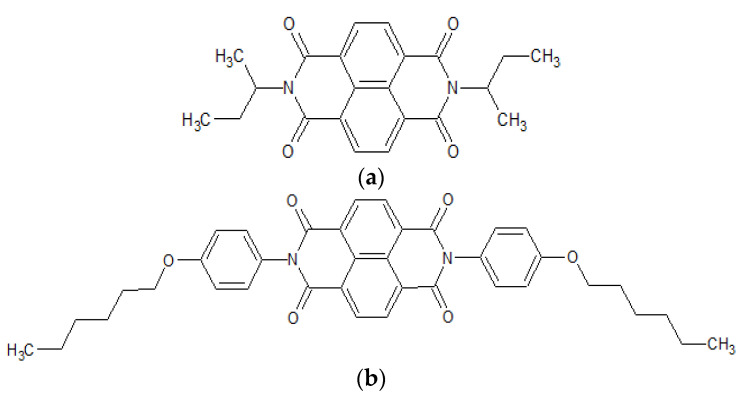
The structural formulae of *N*,*N*′-bis(*sec*-butyl)-1,4,5,8-naphthalenetetracarboxylic acid diimide (NDI-s-Bu) (**a**) and *N*,*N*′-bis(4-n-hexyloxyphenyl)−1,4,5,8-naphthalenetetracarboxylic acid diimide (NDI-4-n-OHePh) (**b**).

Chemical formulae of NDI-s-Bu and NDI-4-n-OHePh are depicted in Figure 1. Their preparation methods, including final products purification and spectroscopic identification as well as elemental analyses, can be found in references [17,22].

Films of NDI-s-Bu were deposited by spin-coating from solutions in chloroform (20 mg of NDI-s-Bu dissolved in 1 mL of chloroform). The exact procedure was as follows: (i) ITO translucent substrates (15 × 20 mm) were first cleaned twice for 10 min in acetone and twice for 10 min in isopropanol baths and then dried in a stream of dry nitrogen; (ii) in the next step they were rinsed for 5 min with chloroform in an ultrasonic cleaner; (iii) the substrates were then dried and placed in a glove box; (iv) chloroform solutions of NDI-s-Bu were then spin-coated on their surface for 30 s using a speed of 1000 revolutions per minute.

This procedure could not be applied to NDI-4-n-OHePh because fast crystallization of this compound prevented from the formation of a uniform layer. Thin layers of NDI-4-n-OHePh were then fabricated by evaporation under vacuum at a rate of 2 nm/s.

To ensure good electrical contact with the ITO electrodes surfaces, selected parts of the layers were washed off with chloroform-soaked sticks and then 100 nm thick aluminum injecting electrodes (Al) of appropriate shape were evaporated through a metal mask. The last fabrication step involved annealing the resulting layer with electrodes attached to it at 70 °C for 15 min.

### 2.2. Electrical Measurements

DC tests for diimides layers sandwiched between the ITO and Al electrodes were carried out in an inert atmosphere of a glove box, with the goal to avoid oxygen and water trapping effects on the electrical transport in the studied layers [23,24]. The thicknesses of the prepared layers, measured using a Bruker DEKTAK XT needle profilometer, were *L_Bu_* = 310 nm and *L_OH_* = 220 nm for NDI-s-Bu and NDI-4-n-OHePh, respectively. A 2410C Keithley multimeter was used as a voltage source.

The obtained current–voltage characteristics were analyzed by applying the drift–diffusion currents for electron-only devices theory [25]. Molecular densities of the investigated materials (*N_m_*) were estimated according to formula:

(1) $$N_{m}=\frac{\rho N_{AV}}{M}$$

where *M* is the molar mass, *N_AV_* is the Avogadro constant and *ρ* is the density of solid-state material. *ρ* values were determined on the bases of crystallographic data presented in [17] and [22] since the deposited layers were highly crystalline.

### 2.3. Quantum Chemical Calculations

The FT calculations were performed with the aim to predict the bandgap and molecular electrostatic potential map for the investigated materials using the Gaussian 09 package with exchange–correlation functional B3LYP and 6-311 + G(d,p) basis set for the optimization of the molecules.

## 3. Results and Discussion

In view of any application of organic semiconductors in devices exploiting the transport of n-type charge carriers, the EA value and the position of the LUMO level, related to each other via Koopmans theorem, are of crucial importance. For the calculation of EA and IP, we have followed the procedure proposed by Sworakowski et al. [26], taking the onset of the first reduction potential and the onset of the first oxidation potential vs. Fc/Fc+ couple. This procedure leads to EA = −3.56 eV for NDI-s-Bu. EA of NDI-4-n-OHePh is higher (−3.71 eV), reflecting weak electron-withdrawing properties of the aryl substituent as compared to the alkyl one. The obtained EA values indicate that NDI-4-n-OHePh molecules are better electron acceptors than oxygen traps, whereas NDI-s-Bu ones are on the borderline [23,24].

NDI-s-Bu is very resistant to oxidation, and its oxidation potential could not be determined by cyclic voltammetry because it exceeded “the potential window” of the electrolyte used [22]. Oxidation potential onset of NDI-4-n-OHePh is 1.27 V vs. Fc/Fc^+^ [17] which yields a very high IP value of 6.25 eV, and by applying the Koopmans theorem, a very low-lying highest occupied molecular orbital (HOMO) level.

It is instructive to compare the obtained experimental values of EA (LUMO) and IP (HOMO) energies with those predicted by DFT calculations (see Table 1).

DFT calculations properly predict significantly lower-lying HOMO levels for NDI-s-Bu, which is also confirmed experimentally by its unmeasurable IP value. Experimental values of EAs of both diimides are similar; DFT calculations also predict similar values of LUMO levels; however, the trend is inverted.

In Table 2, projections of HOMO and LUMO orbitals of both diimides are presented. For NDI-s-Bu, HOMO and LUMO orbitals are not separated in space. They both are located in the aromatic part of the molecule, extending to the imide carbonyl groups. Significantly different frontier orbitals distribution can be encountered for NDI-4-n-OHePh. A clear spatial separation of HOMO and LUMO can be seen here, with LUMO frontier orbitals distributed within the dimide part of the molecule, whereas HOMO orbitals are located on the aryl substituent, being extended to the alkoxy oxygen. This distribution reflects electron-accepting properties of the diimide core and electron-donating ones of the alkoxy substituent. Thus, in the case of this diimide charge, separation should occur more facilely.

**Figure 2:**
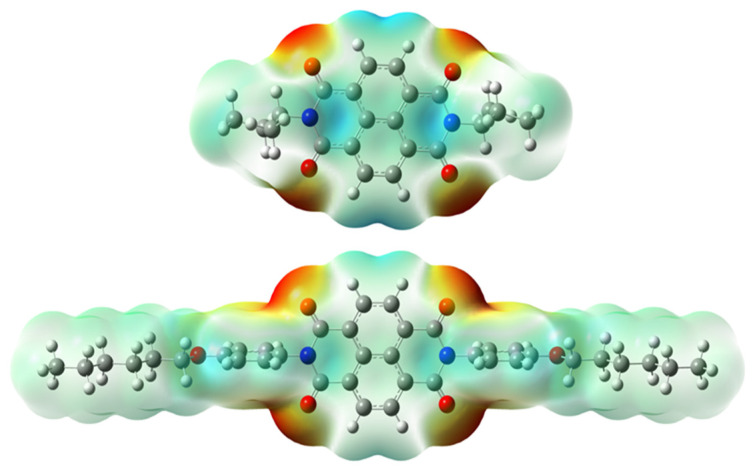
Calculated molecular electrostatic potential surface for NDI-s-Bu (**top**) and NDI-n-OHePh (**bottom**).

Figure 2 presents the mapped molecular electrostatic potential surface, illustrating the electrostatic potential of the rich and poor electron cloud in the molecule. The red color represents the most negative, whereas blue is the most positive parts of the molecule. Other spectral colors represent intermediate values. The color code of the maps is in the range between −3.88 × 10^−2^ a.u. (deepest red) and 3.88 × 10^−2^ a.u. (deepest blue) for NDI-s-Bu and −5.27 × 10^−2^ a.u. (deepest red) to 5.27 × 10^−2^ a.u. (deepest blue) for NDI-4-n-OHePh. For NDI-s-Bu, the red regions in the cloud are located around the carbonyl groups, whereas the blue color is found around the imide heterorings and aromatic rings of the core. A similar color distribution is found in the case of NDI-4-n-OHePh; one should, however, note distinct blue color around the alkoxy oxygen extending to the aromatic ring. The distribution of the electron cloud confirms the more pronounced donor/acceptor character of NDI-4-n-OHePh as compared to NDI-s-Bu, caused by weakly electron-donating alkoxy substituents.

Extensive literature search combining experimental and DFT data clearly indicates that arylene diimides are predominantly electron conductors [10,11,12]. This feature is also unequivocally demonstrated by the DFT calculations presented in this research.

**Figure 3:**
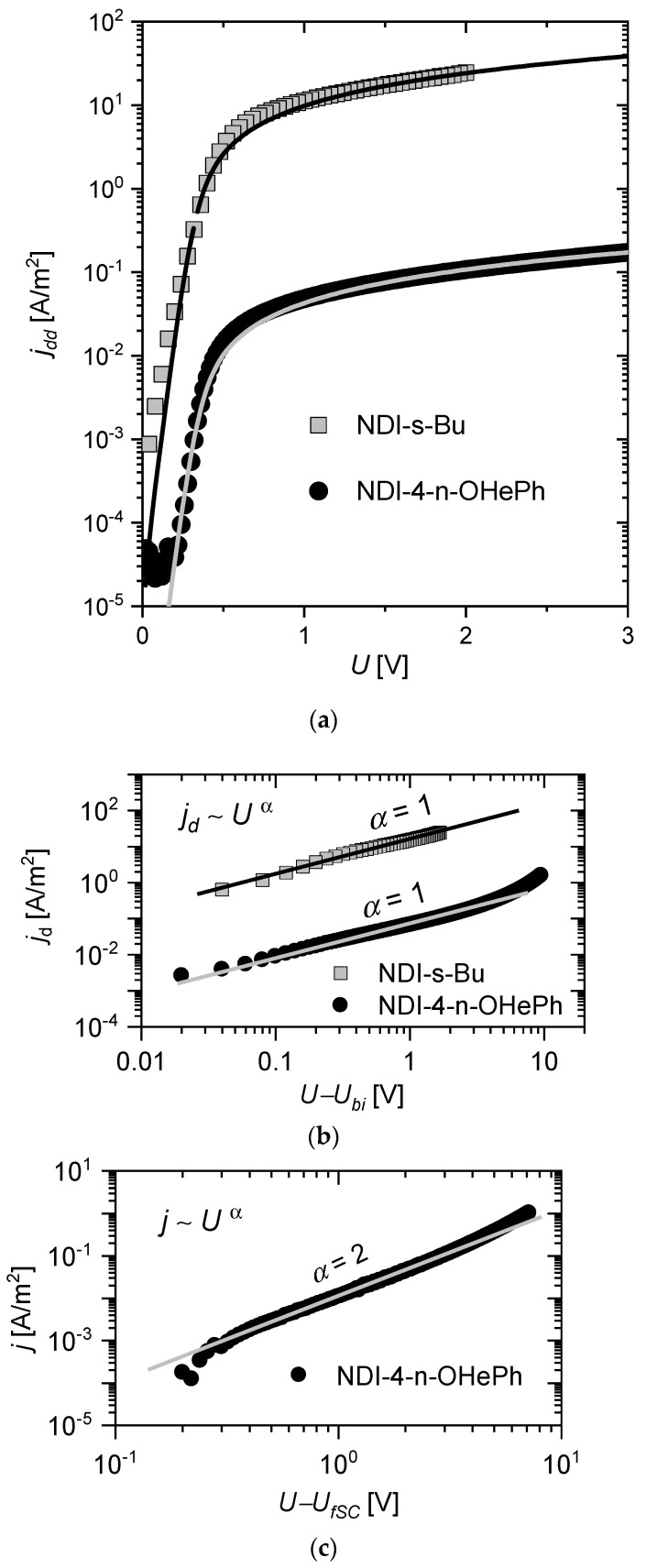
Current-voltage characteristics for electron-only devices based on naphthalene diimides NDI-s-Bu and NDI-4-n-OHePh. The straight lines mark the best fit of the driftdiffusion current model to the current-voltage characteristics (**a**); drift current for bias higher than built-in voltage *U_bi_* (**b**); space-charge-limited current in NDI-4-n-OhePh for bias higher than *U_fSC_* voltage (**c**). The slope of the characteristic to the abscissa (straight lines) (**b**,**c**).

If ITO and Al are selected as electrodes, sandwiches of the following ITO/naphthalene diimide/Al configuration operate as electron-only devices. Current/voltage characteristics of ITO/NDI-s-Bu/Al and of ITO/NDI-n-OHePh/Al devices are presented in Figure 3.

**Figure 4:**
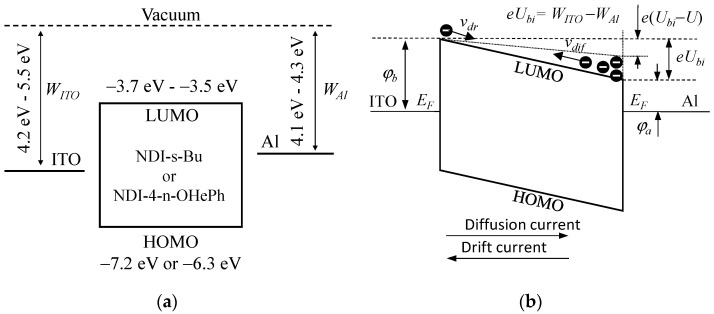
Energies of the HOMO and LUMO levels for the tested naphthalene dimides and the ranges of the ITO and Al electrode work function taken from literature reports; (**a**) energy diagram for electron-only device with two blocking electrodes in thermal equilibrium and upon applying negative bias voltage *U* (dotted line). (**b**) The energy barriers for the injected electrons from the electrodes are *φ_b_* and *φ_a_*. The symbol *U_bi_* denotes the built-in voltage, and the arrows indicate the directions of the electron velocity and the directions of the drift (*ν_d_*) and diffusion (*ν_dif_*) currents.

As already mentioned, the obtained experimental data were analyzed using the classical drift–diffusion current model elaborated by Schottky [27] for a metal contact on a doped inorganic semiconductor. We started the analysis with the consideration of energy relations in the fabricated devices. Figure 4a shows the energy diagram of the tested devices where energy levels of HOMO, LUMO of the organic semiconductors and the work function of the ITO and Al electrodes are indicated. At the electrode/diimide junction, energy barriers exist for the injection of electrons from the ITO (*φ_b_*) and the Al (*φ_a_*) electrodes to the semiconductor.

The electrically short-circuited system is shown in Figure 4b. After reaching the thermodynamic equilibrium of the system, the Fermi levels (*E_F_*) of the electrodes are equalized and the built-in field voltage (*U_bi_*) is created inside the semiconductor.

The model does not take into account the phenomenon of band bending [28,29], which, in the case of one ohmic electrode, strongly influences the conductivity when the samples are polarized with a voltage *U* close to and above *U_bi_* (*U ≈ U_bi_*) [30]. In the case under consideration, the barriers *φ*_a_ and *φ_b_* are so high in relation to the thermal energy *k_B_T* (*k_B_* is the Boltzmann constant and *T* is the temperature) that charge carriers cannot easily diffuse from the electrode into the semiconductor.

Based on the above discussion, it was assumed that in the system under consideration, the built voltage is equal to the difference of work functions of the electrodes (*U_bi_* = *W_ITO_* − *W_Al_*). The analysis did not take into account the effect of electron trapping (mainly by oxygen and water molecules) [31,32], assuming that sample preparation and measurements of current–voltage characteristics were made in glove boxes in a nitrogen atmosphere without oxygen and air access.

The drift–diffusion current model developed for metal–insulator–metal (MIM) structures assumes that inside the semiconductor, the density of free charge carriers is negligibly small, and after applying the potential to the electrodes, the electron energy *E*(*x*) is a linear function of the distance (*x*) from the injecting contact electrode [29]. In the presented case, it can be written as follows:

(2) $$E\left(x\right)=\varphi_{b}+\frac{\left[eU-\left(\varphi_{b}-\varphi_{a}\right)\right]}{L}x$$

A detailed description of such currents for devices consisting of a hole conducting organic semiconductor layer sandwiched between one ohmic and a second blocking can be found in the literature [30,33]. This model was also used to analyze the currents in pure, undoped organic semiconductors exhibiting electron-only transport [34,35]. In this work, a similar analysis was performed for the tested devices, but with both non-ohmic electrodes. The following boundary conditions were assumed:

(3) $$\begin{array}{l} n\left(0\right)=N_{t}exp\left(-\frac{\varphi_{b}}{k_{B}T}\right),\;\;E\left(0\right)=\varphi_{b,}\\ n\left(L\right)=N_{t}exp\left(-\frac{\varphi_{a}}{k_{B}T}\right),\;\;E\left(L\right)=eU+\varphi_{a}, \end{array}$$

where *n*(0) and *n*(*L*) are the electron densities, *E*(0) and *E*(*L*) are energy values of electrons near electrodes and *N_t_* is the density of transport states available for electrons.

After the electrodes are short-circuited, the charges injected from the electrodes drift in the direction determined by the built-in electric field and, at the same time, moves from the electrodes into the layer as a result of diffusion. The generated drift and diffusion currents have the same values but opposite directions (Figure 4b), and no current flows in the external circuit. If the voltage *U* ≤ *U_bi_* is applied to the electrodes, the total density of electrons increases, and then diffusion currents also increase. Initially, for *U* ≤ *U_bi_*, the current is dominated by the diffusion of the charge carriers while vanishing the drift current. In the case of *U* = *U_bi_*, the diffusion current reaches its maximum value, and the drift current is equal to zero. When the applied voltage exceeds the built-in voltage, the drift current changes direction and gradually becomes much higher than the diffusion current.

The calculations assumed that the mobility of electrons (*μ_e_*) in NDI-s-Bu and in NDI-4-n-OHePh does not depend on the electric field strength, and the mobility of charge carriers is related to the diffusion constant by Einstein’s formula *eD* = *μek_B_T* [36,37]. In this case, the current density (*j_dd_*) is given by the equation [30]:

(4) $$j_{dd}=-\mu_{e}kT\left(\frac{dn\left(x\right)}{dx}+\frac{n\left(x\right)}{kT}\frac{dE\left(x\right)}{dx}\right),$$

After substituting *y*(*x*) = *n*(*x*)exp(*E*(*x*)/*k_B_T*), Equation (4) can be converted to the form:

(5) $$j_{dd}=-\mu_{e}k_{B}Tn\left(x\right){exp\left(\frac{E\left(x\right)}{k_{B}T}\right)|}_{0}^{L}{\left[\int_{0}^{L}exp\left(\frac{E\left(x\right)}{k_{B}T}\right)dx\right]}^{-1}$$

where *n*(*x*) is the electron density, *E*(*x*) = *eV*(*x*) is their energy (*V*(*x*) is the electric potential at a distance *x* from the injecting contact) and *L* is the thickness of the layer.

The solution of Equation (5), taking into account the boundary conditions given by Equation (3), is as follows:

(6) $$j_{dd}=\frac{e\mu_{e}N_{t}\exp\left(-\frac{\varphi_{a}}{k_{B}T}\right)}{L}\left[\frac{\left(\varphi_{b}-\varphi_{a}\right)}{e}-U\right]\frac{\left[\exp\left(\frac{eU}{k_{B}T}\right)-1\right]}{\exp\left(\frac{e\varphi_{b}-\varphi_{a}}{k_{B}T}\right)-\exp\left(\frac{eU}{k_{B}T}\right)},$$

From the mutual relations between the LUMO levels and the work function of the electrode shown in Figure 4b, it follows that the built-in field voltage is *U_bi_* = (*φ_b_* − *φ_a_*)/*e*; then, Equation (6) can be written as:

(7) $$j_{DD}=\beta \left(U-U_{bi}\right)\frac{\left[1-\exp\left(-\frac{eU}{k_{B}T}\right)\right]}{\left[1-\exp\left(-\frac{e\left(U-U_{bi}\right)}{k_{B}T}\right)\right]}$$

where *β* = (e*μ_e_N_t_/L*)exp(*eφ_a_*/*k_B_T*).

The value of the built-in field voltage can be estimated from the work function of the ITO and Al electrodes (see Figure 4). Thus, Equation (7) contains only one unknown parameter *N* of the model. It can be relatively easily extracted after fitting the curve predicted by Equation (7) to the experimental data. This is, however, a delicate matter, since it is known that work function for ITO deposited by various methods on different substrate ranges from *E_wITOmin_* = 4.2 eV to *E_wITOmax_* = 5.5 eV [38], and for aluminum from *E_wAlmin_* = 4.06 eV to *E_wAlmax_* = 4.26 eV [39]. It follows that the built-in field voltage *U_bi_* in the tested compounds may vary in a wide range from (*E_wITOmin_*–*E_wAlmax_*) to (*E_wITOmax_*–*E_wAlmin_*) (0 eV ≤ *U_bi_* ≤ 1.4 eV). Therefore, at least two parameters had to be determined to extract the physical quantities describing the tested samples.

The best fits of this model (dashed lines in Figure 3a) to the experimental data were obtained for built-in voltages *U_biBu_* = 0.32 V and *β_Bu_* = 14.4 A/m^2^ for ITO/NDI-s-Bu/Al, and *U_biOH_* = 0.34 V and *β_OH_* = 6.5 × 10^−2^ A/m^2^ for ITO/NDI-4-n-OHePh/Al devices.

For a sufficiently high voltage *U >> U_bi_*, the component related to the charge carrier diffusion current *μ_e_k_B_Tdn*(*x*)/*dx* in Equation (4) can be omitted, and, then, the drift current (*j_d_*) prevails. In this case, the expressions exp[−(*eU*/*k_B_T*)] in the numerator and exp[−(*eU*/*k_B_T*)] in the denominator of Equation (7) are much less than unity, and the current approximates linearly to (*U*—*U_bi_*):

(8) $$j_{d}\approx \frac{e\mu_{e}N}{L}\left(U-U_{bi}\right)$$

The current–voltage characteristics for the tested samples shown in Figure 3b are linear in the voltage range from 0.7 V to 2 V for NDI-s-Bu and from 0.9 V to 2.8 V for NDI-4-n-OHePh. This means that for voltages higher than *U_bi_*, the model assumptions given by Equation (3), are still valid. This compatibility of the experiment with the model also proves that the phenomenon of mobility enhancement due to the density of states filling [40,41] can be neglected, and, as a result, the mobility of charge carriers remains constant.

In the case of NDI-4-n-OHePh, for the voltage higher than 2.8 V, a space charge is formed, and the electric field distribution in the semiconductor is modified. The assumption of the drift–diffusion current model consistent with Equation (3) weakens with increasing voltage, and the drift current *j_d_* is gradually replaced by the space-charge-limited current (*j_SCLS_*). For *U* close to voltage forming space charges (*U_fSC_* = 2.8 V), the total current density (*j*) can be described as:

(9) $$j=j_{d}+j_{SCLC}$$

where *j_SCLS_* is described by the Mott–Gurney equation [42] in the form:

(10) $$\;\;\;\;\;\;\;j_{SCLC}=\frac{9}{8}\frac{\mu_{e}\varepsilon_{r}\varepsilon_{0}}{L^{3}}{\left(U-U_{fSC}\right)}^{2}.$$

where *ε* is the dielectric constant, *ε_0_* is the electric constant.

For the ITO/NDI-4-n-OHePh/Al, it was possible to observe the SCLC (see Figure 3c). In the voltage range from 0.5 V to 3 V (*U–U_fSC_*), the relationship between current and voltage can be described by a square function. After fitting the theoretical plot (9) to the experimental data (with the dielectric constant *ε* = 3), the value of the electron mobility was estimated as *μ_eOH_* = 4.6 × 10^−6^ cm^2^V^−1^s^−1^.

The DFT calculations show that the energies of the LUMO-1, LUMO-2, LUMO-3 levels in both tested naphthalene diimide derivatives are higher than the LUMO-0 level by 1.7 eV and more (Table 1), and it is highly probable that in the solid state, the density transport is equal to the molecular density of investigated materials *N_m_*. These parameters were estimated by performing elementary calculations using Equation (1). The density of the crystalline form of NDI-s-Bu (*ρ_Bu_* = 1361 g/cm^3^) was determined on the basis of crystallographic data presented in [22]. In the case of NDI-4-n-OHePh, the density derived from crystallographic data was *ρ_OH_* = 1302(5) g/cm^3^ [17]. The resulting densities of transport centers were: *N_tBu_* = 2.1 × 10^27^ m^−3^ and *N_tOH_* = 1.2 × 10^27^ m^−3^.

Considering the experimental and calculation results presented in Table 1, it can be considered that the LUMO levels of both tested compounds, i.e., NDI-4-n-OHePh and NDI-s-Bu are very similar. It can therefore be assumed that the barriers *φ_a_* and *φ_b_* the injection of electrons from the electrodes have the same values for both organic semiconductors: *φ_a_* = 0.40 eV and *φ_b_* = 0.73 eV. The built-in field voltage *U_bi_* = 0.33 eV was adopted for the calculations and the following formulae were used: *φ_a_* = ln[*eμ_eOH_N_tOH_*/(*Lβ_OH_*)] and *φ_b_* = *U_bi_*+*φ_a_*. Based on this assumption, the electron mobility for NDI-s-Bu was also estimated on the basis of the formula:

(11) $$\mu_{eBu}\;=\;\mu_{eOH}\frac{\beta_{eBu}L_{OH}\rho_{OH}M_{Bu}}{\beta_{eOH}L_{Bu}\rho_{Bu}M_{OH}}$$

The value *μ_eBu_* = 4.3 × 10^−4^ cm^2^V^−1^s^−1^ of mobility was obtained.

The mobility quotient *μ_eBu_*/*μ_eOH_* ≈ 100 is in agreement with the results reported in the literature. In particular, the mobility of electrons in the OFET configuration measured for thin layers of NDI-s-Bu was *μ_eBuFET_* = 1.6 × 10^−1^ cm^2^V^−1^s^−1^ [43], whereas in the case of NDI-4-n-OHePh layers it reached *μ_eOHFET_* = 4.0 × 10^−3^ cm^2^V^−1^s [17]. High mobility reported for NDI-s-Bu was a result of highly ordered supramolecular organization in which planar naphthalene diimide cores formed oriented columns parallel to each other. High ordering of molecules facilitates the transport of charge carriers in the transistor channel and contributes to increasing the mobility of the charge carriers by orders of magnitude in relation to the mobility in layers with no ordering [44]. In such anisotropic systems, π-stacked molecules favored the transport of electrons along molecular columns oriented perpendicular to the drain and source electrodes in the transistor channel. Supramolecular order also contributed to an increase in the density of available transport states, which resulted in high drain currents being recorded. The value of the mobility of the charge carriers, determined from the current–voltage characteristics of the field-effect transistors, is an increasing function of the charge carrier density [45,46]. The aforementioned factors significantly influenced the enhancement of the electron mobility. However, the transport of electrons in comparable systems containing ordered layers of naphthalene diimides was still over 100 times better in the NDI-s-Bu layer than in the NDI-4-n-OhePh one.

For the LUMO level energy from 3.5 eV to 3.7 eV, it was estimated that the work functions of ITO and Al should have been in the range from 4.3 eV to 4.5 eV and from 3.9 eV to 4.1 eV and, respectively (see Table 3). These values are in good agreement with the values of ITO and Al work functions most often used in the analysis of the operating organic electronics devices [47,48,49]. Thus, the results reported here can be considered as a strong argument for the correctness of the drift–diffusion current model adopted for the analysis of the presented experimental results.

The observed discrepancy between the model and the experiment deserves a comment. The current–voltage characteristics showed a clear, linear current–voltage dependence for voltages higher than the build-in voltage. However, the curves drawn on the basis of the parameters *β* and *U_bi_* estimated on the basis of Equation (7) significantly differed from the experimental points for voltages lower than *U_bi_*. The calculated current increased too slowly with increasing voltage, and the estimated built-in field voltage of 0.18 V was too low, which resulted in an aluminum work function lower than 3.9 eV, a value that could not be accepted. Often, the concept of band-binding caused by the migration of charge carriers from the ohmic electrode to the semiconductor is introduced into the model drift–diffusion current [28,30,50]. The introduction of an additional potential barrier causes the values of the determined parameters to be more realistic. It should be noted, however, that the boundary conditions adopted in this work for the tested electron-only devices and the solution of the drift–diffusion current equation are mathematically identical as in the case of systems with an injected ohmic electrode and band-bending effect (see Equation (3) in [50]). The introduction of such a barrier in the considered model would lead to a reduction of the built-in field voltage and thus to an even lower value of the determined work function for ITO and Al electrodes. It would also lead to a decrease in the built-in field voltage and thus to an even lower value of the determined work function of the ITO and Al electrodes. It follows that the commonly used, often successfully, drift–diffusion current model still requires taking into account additional phenomena affecting the diffusion current in devices containing organic semiconductors with two non-ohmic electrodes.

## 4. Conclusions

The presented research has shown that naphthalene diimide N-functionalized with alkyl and aryl substituents, namely NDI-s-Bu and NDI-4-n-OHePh are strongly unipolar, being characterized by good electron transport properties and essentially negligible hole transport. These properties could be somehow predicted by calculated measured HOMO and LUMO energies and electrochemically determined electron affinity (EA) and ionization potential (IP) values, showing that the studied molecules were easy to reduce (inject electron) and difficult to oxidize (inject hole).

The current–voltage characteristics obtained for sandwich-type devices ITO/NDI-s-Bu/Al and ITO/NDI-4-n-OHePh/Al were analyzed for low voltages in terms of drift–diffusion current model and for higher voltage using the Mott-Gurney model of space-charge-limited current. Fitting the experimental data to these models yielded the electron mobility for NDI-s-Bu and NDI-4-n-OHePh. In Table 3, the electrical transport data derived from experimental current–voltage characteristics are collected.

It was also shown that the use of two blocking electrodes and the injection of charge carriers (instead of one ohmic and the other blocking) allowed for a reliable analysis of the experimental current–voltage characteristics in electron-only devices using the original version of the drift–diffusion–current model. In this case, satisfactory results were obtained without the necessity of the consideration of band bending at the electrodes. However, a certain discrepancy between the model and the experimental results in the diffusion current for voltages lower than the built-in field voltage was noted.

## Figures and Tables

**Table 1:** Experimentally measured IP and EA and theoretically predicted HOMO and LUMO energies.

	IP (eV)	EA (eV)	HOMO (eV)	LUMO (eV)
NDI-s-Bu	- ^1^	−3.56	−7.17	−3.54
NDI-4-n-OHePh	6.25	−3.71	−6.28	−3.43

^1^ Unmeasurable in the electrolyte used.

**Table 2:** Calculated projections of HOMO, LUMO and energetically nearest orbitals of both diimides.

	LUMO	*E* [eV]	HOMO	*E* [eV]
NDI-s-Bu	+3	−1.19	0	−7.17
	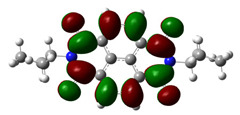		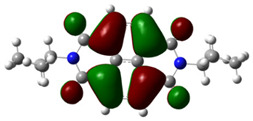	
	+2	−1.45	−1	−7.70
	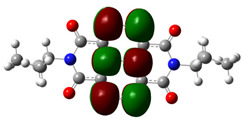		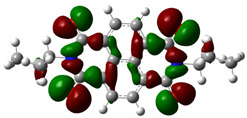	
	+1	−1.83	−2	−7.72
	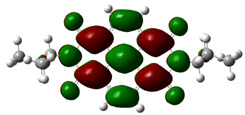		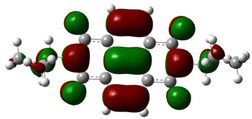	
	0	−3.54	−3	−7.84
	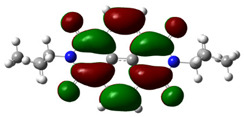		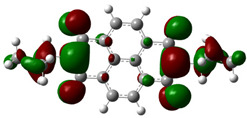	
NDI-4-n-OHePh	+3	−1.03	0	−6.28
	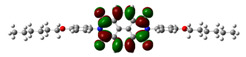		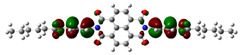	
	+2	−1.38	−1	−6.29
	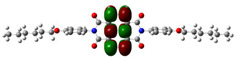		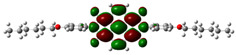	
	+1	−1.76	−2	−7.06
	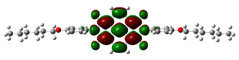		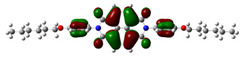	
	0	−3.43	−3	−7.18
	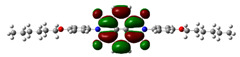		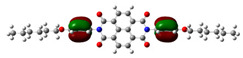	

**Table 3:** Experimentally determined electron mobility for NDI-s-Bu and for NDI-4-n-OHePh and work function of ITO and Al electrodes.

Method	Parameter	NDI-s-Bu	NDI-4-n-OHePh
Experiment	*μ_e_* [cm^2^V^−1^s^−1^]	4.3 × 10^−4^	4.6 × 10^−6^
	*W_ITO_* [eV]	4.5 ** (4.3 *)	
	*W_Al_* [eV]	4.1 ** (3.9 *)	

The calculations were made for the energy of LUMO taken from Table 2 (~3.5 eV) * and determined experimentally, taken from literature reports **.

## Data Availability

The data presented in this study are available on request from the corresponding author.
